# Detailed evaluation of the upper airway in the Dp(16)1Yey mouse model of Down syndrome

**DOI:** 10.1038/s41598-020-78278-2

**Published:** 2020-12-07

**Authors:** Tatsunori Takahashi, Noriaki Sakai, Tomonori Iwasaki, Timothy C. Doyle, William C. Mobley, Seiji Nishino

**Affiliations:** 1 Sleep and Circadian Neurobiology Laboratory, Department of Psychiatry and Behavioral Sciences, Stanford University School of Medicine, 3155 Porter Drive, Room 2141, Palo Alto, CA 94304 USA; 2 Department of Pediatric Dentistry, Kagoshima University Graduate School of Medical and Dental Sciences, 8-35-1, Sakuragaoka, Kagoshima, Kagoshima 8908544 Japan; 3 The Neuroscience Community Labs, Wu Tsai Neurosciences Institute, Stanford University, 318 Campus Drive, Suite S170, Stanford, CA 94305 USA; 4 Department of Neurosciences, University of California San Diego School of Medicine, 9500 Gilman Drive, La Jolla, CA 92093 USA; 5 Present Address: Department of Medicine, Jacobi Medical Center, Albert Einstein College of Medicine, 1400 Pelham Parkway South, Bronx, NY 10461 USA

**Keywords:** Preclinical research, X-ray tomography

## Abstract

A high prevalence of obstructive sleep apnea (OSA) has been reported in Down syndrome (DS) owing to the coexistence of multiple predisposing factors related to its genetic abnormality, posing a challenge for the management of OSA. We hypothesized that DS mice recapitulate craniofacial abnormalities and upper airway obstruction of human DS and can serve as an experimental platform for OSA research. This study, thus, aimed to quantitatively characterize the upper airway as well as craniofacial abnormalities in Dp(16)1Yey (Dp16) mice. Dp16 mice demonstrated craniofacial hypoplasia, especially in the ventral part of the skull and the mandible, and rostrally positioned hyoid. These changes were accompanied with a shorter length and smaller cross-sectional area of the upper airway, resulting in a significantly reduced upper airway volume in Dp16 mice. Our non-invasive approach, a combination of computational fluid dynamics and high-resolution micro-CT imaging, revealed a higher negative pressure inside the airway of Dp16 mice compared to wild-type littermates, showing the potential risk of upper airway collapse. Our study indicated that Dp16 mice can be a useful model to examine the pathophysiology of increased upper airway collapsibility of DS and to evaluate the efficacy of therapeutic interventions for breathing and sleep anomalies.

## Introduction

Down syndrome (DS) is a chromosomal disorder resulting from the presence of an extra copy of human chromosome 21^1^. Its clinical phenotypes involve multiple organs and are characterized by dysmorphic facial features, congenital cardiovascular abnormalities, and growth retardation^1^. Obstructive sleep apnea (OSA) is a common problem of DS. It is estimated that the prevalence of OSA reaches up to 63% in children with DS^2,3^. Moreover, even those without evidence of OSA have a smaller upper airway^4^. The high prevalence of OSA and narrowed upper airway in DS is attributed to the coexistence of various characteristics related to its genetic abnormality. Midfacial and mandibular hypoplasia is a crucial predisposing factor and restricts the skeletal enclosure around the upper airway^4^. Thus, even a normal-sized tongue works as a relatively large tongue in the constricted skeletal structures and contributes to upper airway narrowing^4,5^. Generalized hypotonia and obesity are also recognized as risk factors for upper airway obstruction associated with DS^6^. The multifactorial nature of OSA poses a challenge to determine the definitive cause in DS individuals, resulting in the high frequency of residual OSA after adenotonsillectomy^7^.

Recently, the application of computational fluid dynamics (CFD) has been validated as a practical and powerful tool for better understanding subject-specific mechanisms of OSA. Previous reports have demonstrated its advantages over anatomical evaluation alone^8–10^. CFD has also been utilized not only to predict the effect of OSA treatments in general populations^11–13^, but also to tailor treatment strategies for DS individuals with OSA^14^. Although CFD has the potential to visualize and stratify multilevel narrowing of the upper airway, CFD has not been used for this purpose in mice. A major obstacle to the upper airway evaluation in live small animals has been that conventional *in-vivo* preclinical imaging modalities cannot provide high-resolution images with a technically feasible method. This results in failure of generating a mouse-specific configuration of the upper airway. In contrast, the latest models of micro-computed tomography (micro-CT) allow fast scanning with low radiation dose and yet provide high spatial resolution. These instruments enable the utilization of CFD for a detailed evaluation of the upper airway of mice in a non-invasive manner.

Many mouse models for DS have been developed to mimic human DS. However, strain-specific differences between the mouse syntenic regions and human chromosome 21 are known. The Ts65Dn mouse is trisomic for about 60% (~ 13 Mb) of the genes orthologous to human chromosome 21 on mouse chromosome 16 and has been most widely used as a mouse model for DS research^15,16^. Yet, the additional chromosome also carries non-syntenic segments on mouse chromosome 17, limiting the utility of this strain^17^. In 2007, Li et al.^18^ generated a new mouse model which is trisomic for all the human chromosome 21 syntenic regions (22.9 Mb) on mouse chromosome 16 and which lacks non-syntenic trisomic segments of mouse chromosome 17. This Dp(16)1Yey (Dp16) mouse is recognized as the most promising model to study pathophysiology and therapeutic interventions.

We hypothesized that DS mice recapitulate craniofacial abnormalities and upper airway obstruction of human DS. This study, thus, aimed to quantitatively characterize the upper airway as well as craniofacial abnormalities including the hyoid position utilizing high-resolution micro-CT imaging in combination with computational modeling of airflows. Our findings represent that Dp16 mice can be a useful model to understand upper airway anomalies or even collapse when additional predisposing factors for OSA coexist.

## Results

### Baseline characteristics and respiratory parameters

Age did not differ significantly between the groups even when the groups were subdivided by sex (Table 1). Similarly, we observed no significant difference in body weight between wild-type littermates (WT) and Dp16 groups (WT = 22.23 ± 2.83 g; Dp16 = 20.40 ± 3.27 g, P = 0.251).

**Table 1:** Baseline characteristics of WT and Dp16 mice.

	WT (N = 8)		Dp16 (N = 8)		*P*
	Mean	SD	Mean	SD	
Age (days)	57	12	58	12	0.934
Male	49	11	49	11	0.974
Female	65	6	66	7	0.873
Weight (g)	22.23	2.83	20.40	3.27	0.251

*WT* wild-type, *Dp16* Dp(16)Yey.

**Figure 1:**
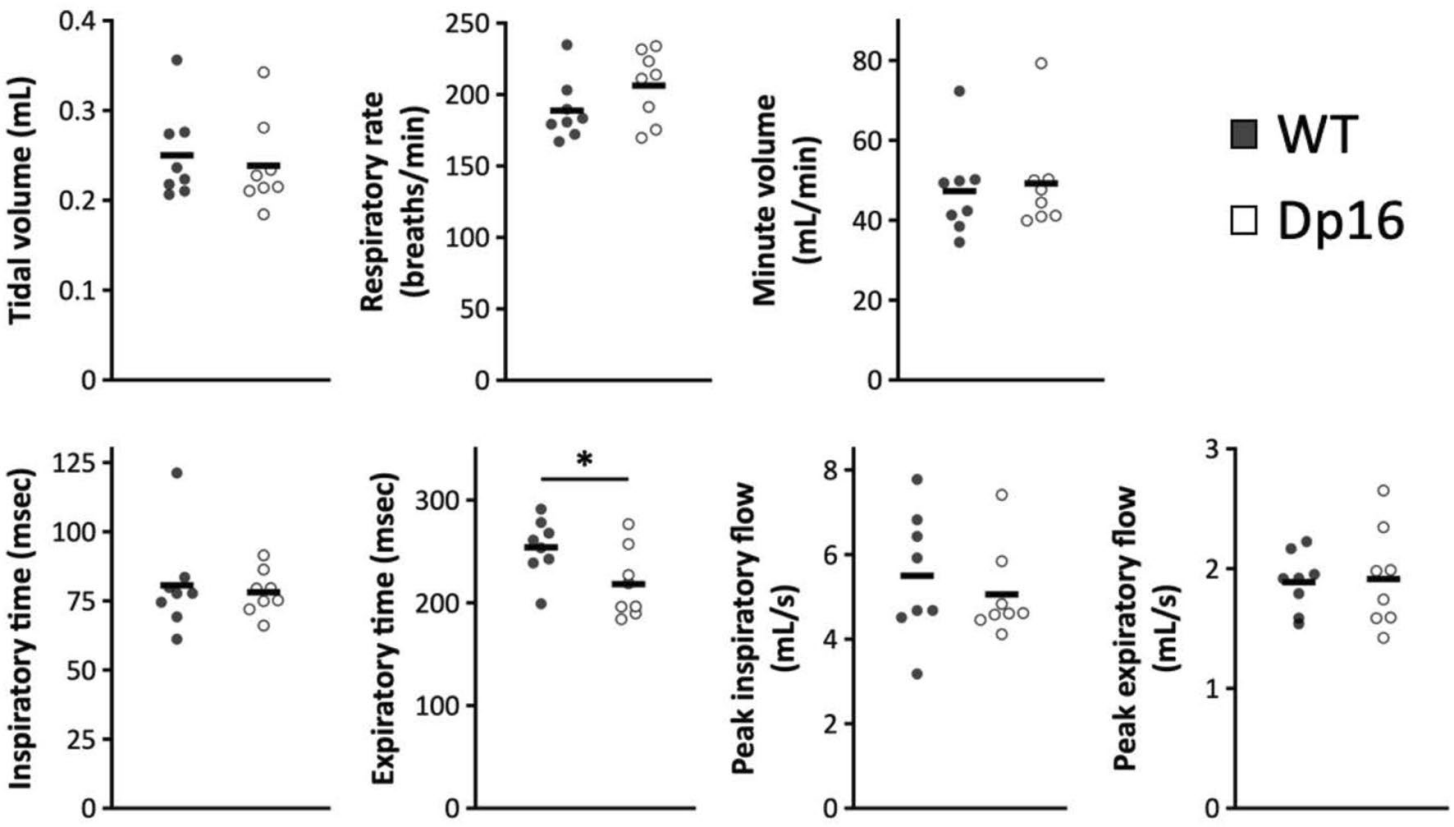
Comparison of respiratory parameters obtained using whole-body plethysmography. Seven parameters were obtained from plethysmography. Values are presented as means ± SD. *P < 0.05.

The results of plethysmography which was performed at room temperature in 21% O_2_ balanced with N_2_ showed significantly lower expiratory time in Dp16 mice (WT = 254.05 ± 28.25 ms; Dp16 = 218.26 ± 33.70 ms, *P* = 0.038) and no difference in inspiratory time between genotypes (WT = 80.61 ± 17.83 ms; Dp16 = 78.12 ± 8.03 ms, *P* = 0.727), resulting in relative prolongation of the inspiratory time for the expiratory time in Dp16 mice (Fig. 1). Other parameters did not differ significantly, indicating that awake Dp16 mice had little or no apparent respiratory abnormalities.

### Craniofacial measurements and the hyoid position

**Figure 2:**
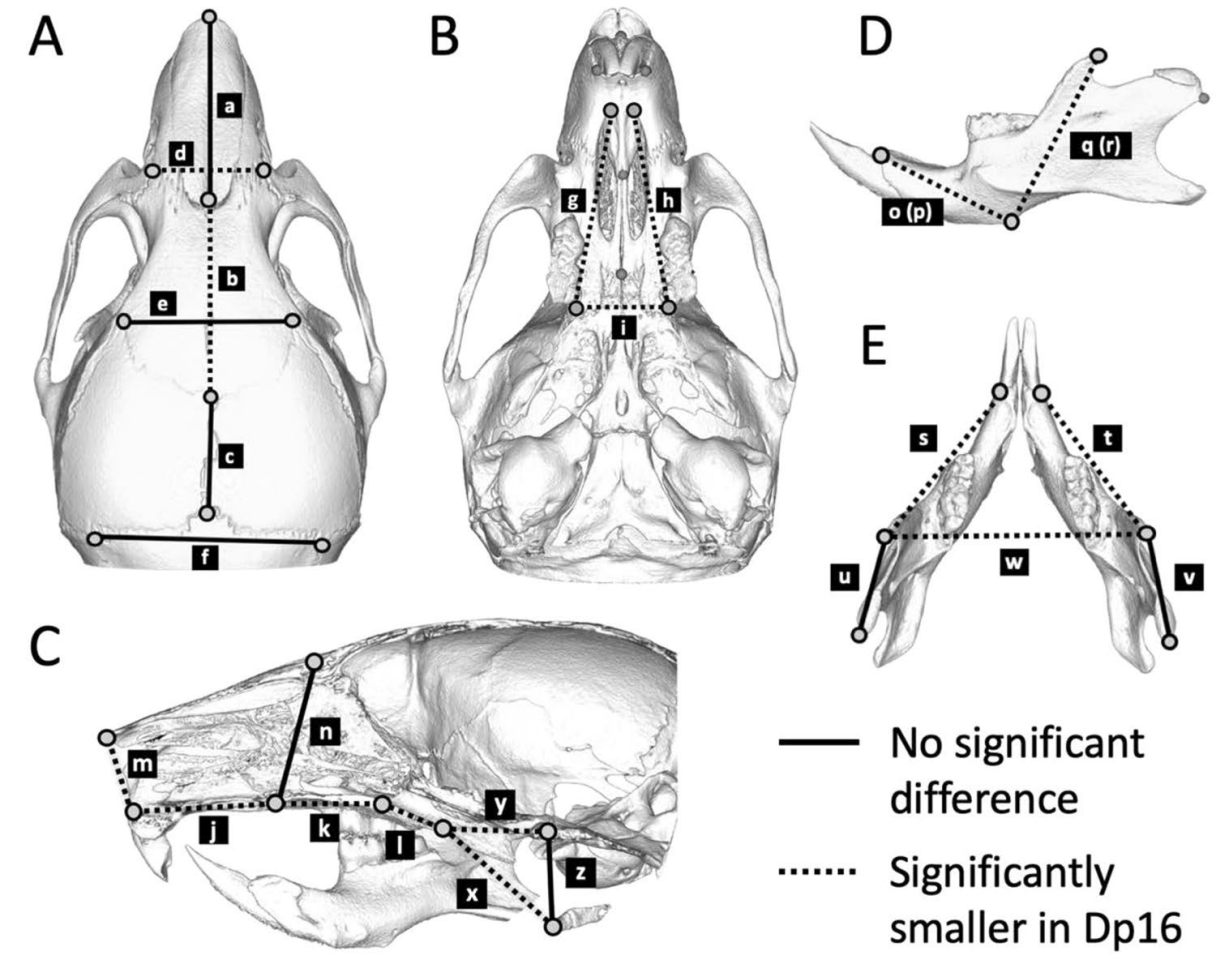
Comparison of craniofacial measurements and the hyoid position. The dorsal (**A**), ventral (**B**) and sagittal (**C**) views of the cranium and the lateral (**D**) and dorsal (**E**) views of the mandible are shown. Solid lines indicate linear distances that are similar between genotypes, while dotted lines represent those significantly smaller in Dp16 mice than WT mice. Values and statistics are summarized in Table 2.

The size of craniofacial bones in Dp16 mice significantly differed compared to that of WT mice (Table 2, Fig. 2). In the measurements of the dorsal aspect of the skull, the frontal bone length in Dp16 mice was significantly smaller (Fig. 2A, b), whereas the nasal and parietal bone lengths showed no significant difference between genotypes (Fig. 2A, a and c). Dp16 mice also demonstrated the shorter width between the left and right intersections of frontal, premaxilla, and maxilla bones (Fig. 2A, d), which could result in a narrow space of the nasal cavity. Moreover, measurements of the ventral aspect of the skull (Fig. 2B,C, g–l) and those of the dorsoventral axis (Fig. 2C, m and n) revealed that almost all linear distances measured were significantly smaller in Dp16 mice when compared to those of WT mice. Similar results were observed in the mandible measurement (Table 2 and Fig. 2D,E), showing all lengths except for the distance between the coronoid process and posterior-most point on the mandibular condyle (Fig. 2E, u and v) were significantly shorter in Dp16 mice. Overall, our results revealed hypoplasia of the cranial base, maxilla, and mandible in Dp16 mice. Similar results were observed regardless of sex (Supplemental Table).

**Table 2:** Craniofacial measurements between WT and Dp16 mice.

	Landmarks (Suppl. Figure 1)	WT		Dp16		*P*
		Mean	SD	Mean	SD	
Skull (Fig. 2A–C)						
Dorsal view						
a	1–3	7.09	0.20	7.03	0.19	0.584
b	3–6	7.93	0.17	7.33	0.20	< 0.001
c	6–9	3.82	0.15	3.69	0.28	0.344
d	2–4	4.20	0.06	3.78	0.05	< 0.001
e	5–7	5.98	0.19	6.00	0.16	0.831
f	8–10	7.88	0.19	7.53	0.27	0.018
Ventral view						
g	11–15	7.41	0.17	6.84	0.07	< 0.001
h	12–16	7.42	0.19	6.79	0.09	< 0.001
i	15–16	3.40	0.04	3.19	0.09	< 0.001
j	13–18	5.27	0.14	4.74	0.26	< 0.001
k	13–14	3.75	0.12	3.37	0.21	0.001
l	14–17	2.11	0.05	1.92	0.08	0.001
Lateral view						
m	1–18	2.66	0.09	2.51	0.05	0.002
n	3–13	4.78	0.11	4.66	0.16	0.125
Mandible (Fig. 2D,E)						
o	19–21	5.10	0.11	4.75	0.15	< 0.001
p	20–22	5.10	0.14	4.66	0.15	< 0.001
q	21–23	6.86	0.13	6.47	0.18	< 0.001
r	22–24	6.92	0.12	6.53	0.18	< 0.001
s	19–23	8.57	0.11	7.92	0.19	< 0.001
t	20–24	8.57	0.19	7.96	0.21	< 0.001
u	23–25	3.93	0.14	3.86	0.17	0.360
v	24–26	3.87	0.12	3.85	0.18	0.766
w	23–24	9.46	0.14	9.06	0.25	0.005
Hyoid position (Fig. 2C)						
x	17–28	4.99	0.12	4.70	0.07	< 0.001
y	17–27	4.18	0.14	3.33	0.19	< 0.001
z	27–28	2.79	0.12	2.79	0.13	1.000

The unit is millimeter.

We further measured the distances between the hyoid, the caudal edge of the hard palate, and the ventral point on the basisphenoid-occipital suture to evaluate rostrocaudal and ventrocaudal dislocations of the hyoid (Figure S1). Dp16 mice exhibited a significantly shorter distance between the hyoid and the hard palate (Fig. 2C, x and Table 2) while there was no dislocation in the ventrocaudal direction (Fig. 2C, z and Table 2), suggesting the hyoid of Dp16 mice is rostrally positioned.

### Cross-sectional area of upper airway

**Figure 3:**
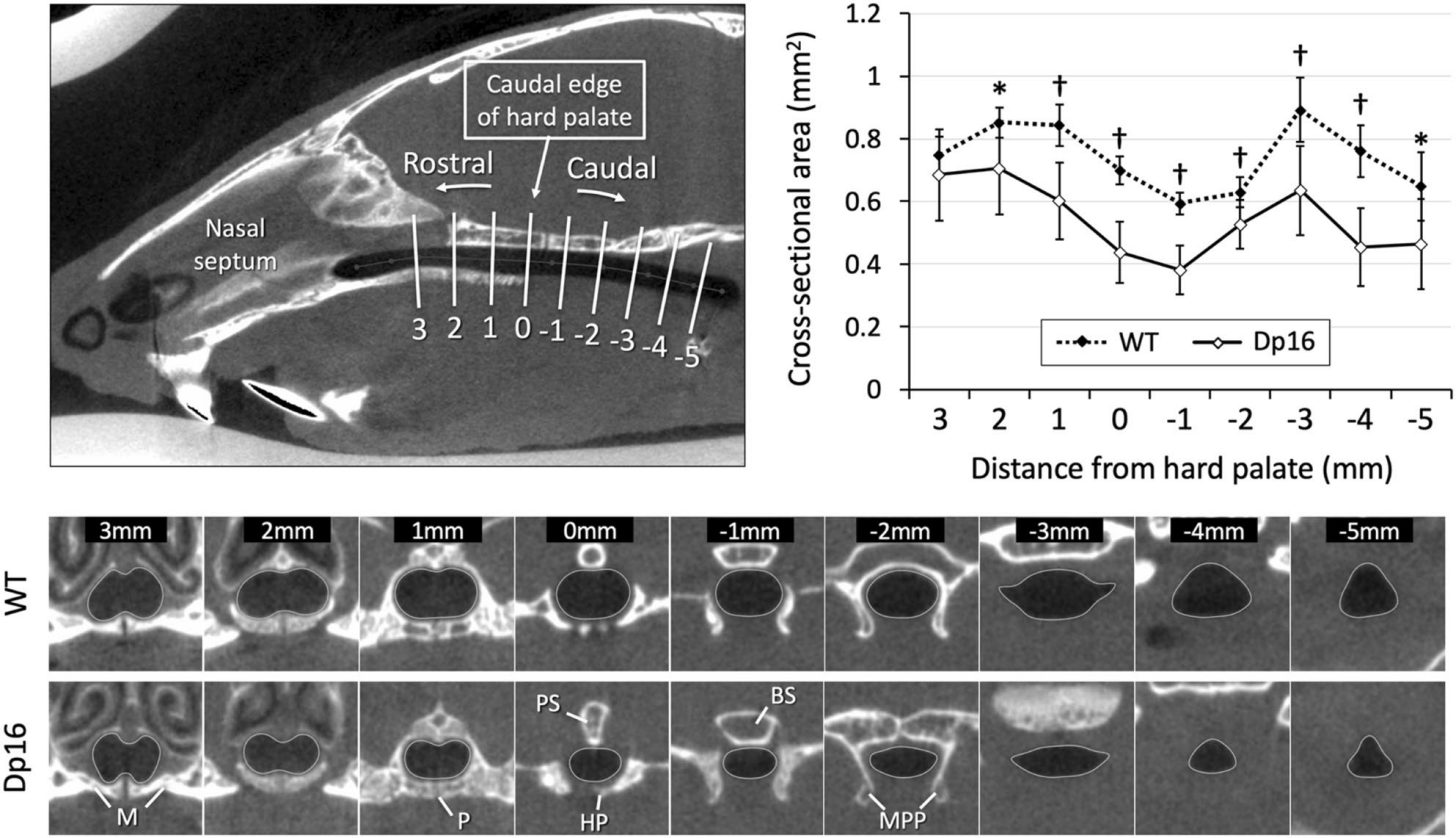
Evaluation of the upper airway and statistical comparison of its cross-sectional areas. (Upper left) The caudal edge of the hard palate was used as a landmark (0). Nine consecutive cross-sectional images perpendicular to the upper airway were obtained at 1 mm intervals. (Bottom) The representative images from a mouse of each genotype are shown. (Upper right) Cross-sectional areas were measured and plotted at each point. Values are presented as means ± SD. *P < 0.05; ^†^*P* < 0.001. *M* maxilla, *P* palatine, *PS* presphenoid, *HP* hard palate, *BS* basisphenoid, *MPP* medial pterygoid plate.

The upper airway morphology was analyzed from the nasopharynx to the epiglottis. The shape and size were not consistent through the airway and were influenced by the surrounding tissue. The nasopharyngeal airway showed a dumbbell-shaped appearance (2 and 3 mm rostral to the hard palate in Fig. 3). The ventral part of the nasopharyngeal airway contacted the bone (i.e., the maxilla) while the other part was surrounded by the tissues constructing the nasal cavity such as the septum or ethmoid turbinates. The airway shape gradually became oval towards the hard palate (from 1 to − 2 mm). The nasopharyngeal airway close to the edge of the hard palate was enclosed by bones such as the palatine and the presphenoid bones. At the transition zone from the hard palate to the soft palate, the airway was surrounded by the presphenoid or basisphenoid bone dorsally and the medial pterygoid plates bilaterally (from 0 to − 2 mm). Beyond the medial pterygoid plates, the surrounding was mainly composed of soft tissues and the airway enlarged laterally, showing a lemon-shaped appearance, followed by a triangular shape near the epiglottis (from − 3 to − 5 mm). With regard to shape, no apparent difference was observed between genotypes.

On the other hand, quantitative analysis revealed that the upper airway was significantly smaller in almost all the areas in Dp16 mice compared to that in WT mice (Fig. 3). The upper airway gradually became narrower and its size reached a nadir at the beginning of the soft palate segment where the medial pterygoid plates bilaterally surround the airway (− 1 mm). Beyond the narrowest point, the upper airway enlarged at 3 mm caudal to the hard palate where the surrounding was mainly composed of soft tissues.

**Figure 4:**
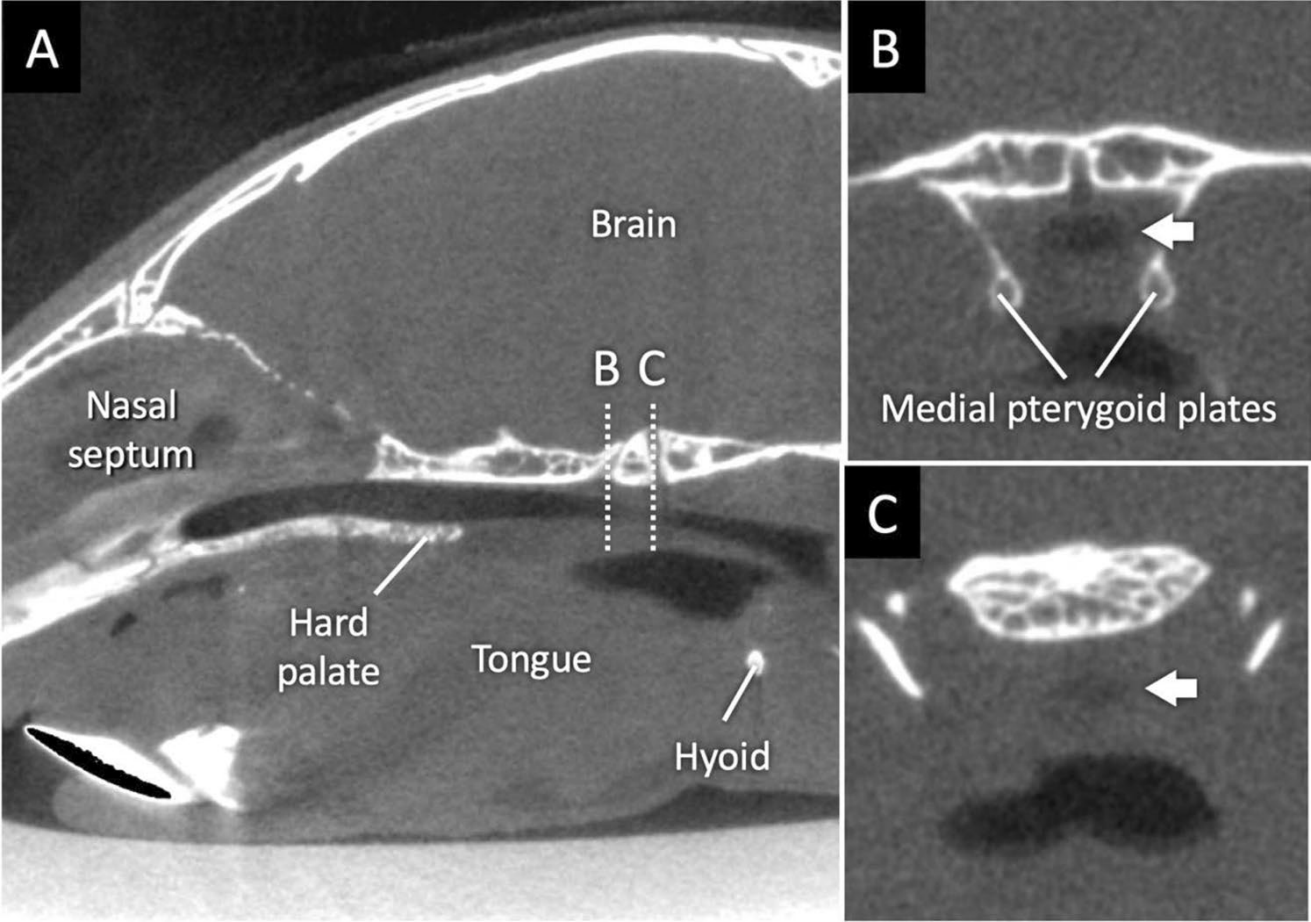
Upper airway collapse in a Dp16 mouse. (**A**) Upper airway collapse was observed in one of Dp16 mice. The mouse was breathing in the prone position during scanning. Dotted lines B and C are severely narrowed points which are surrounded by bones or soft tissues, respectively. Cross-sectional images, (**B**) and (**C**), were obtained from dotted lines B and C, respectively. White arrows indicate the collapsed lumen.

One of the Dp16 mice exhibited a regional upper airway collapse in the soft palate segment (Fig. 4). This finding was most likely due to the isoflurane-induced abnormal respiratory movements of the soft palate and the pharyngeal airway throughout the scanning^19^, but not due to swallowing or persistent obstruction, because blurry contours were limited to these structures. This mouse was rescanned on the following day for quantitative and flow analyses.

### CFD analysis

**Figure 5:**
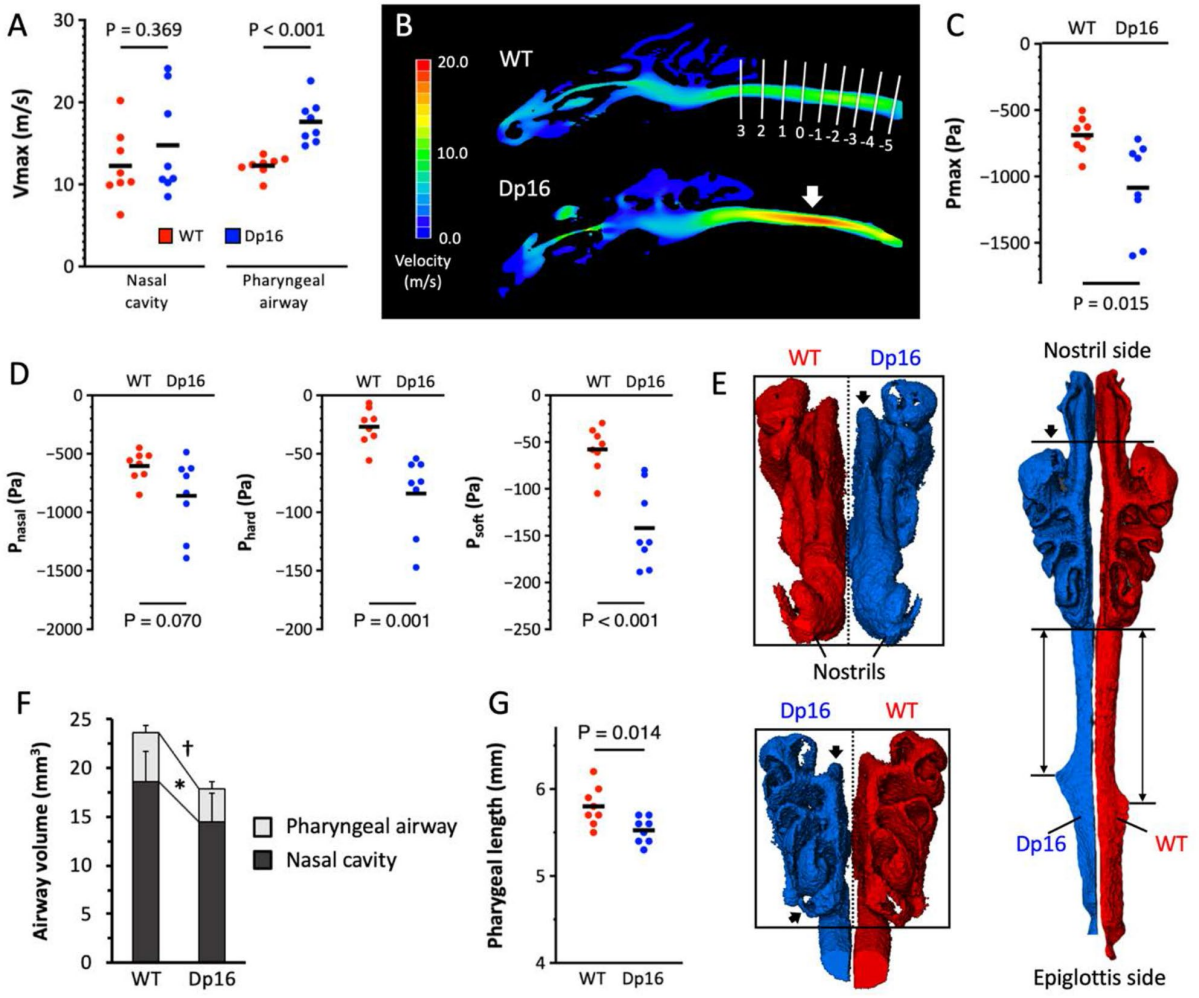
Airflow analyses using computational fluid dynamics. (**A**) Statistical comparisons of the Vmax in the nasal cavity and pharyngeal airway are shown. (**B**) The sagittal slices of the velocity analysis obtained from a representative mouse of each genotype illustrate a notable acceleration in the pharyngeal airway of the Dp16 mouse (46-day-old, male WT mouse weighing 22.7 g vs. 47-day-old, male Dp16 mouse weighing 22.5 g). The location of the highest velocity corresponds with the narrowest part of the upper airway (arrow). White solid lines indicate same lines shown in Fig. 3. (**C**) The Pmax was estimated at the level of the epiglottis. Dp16 mice had a significantly higher negative pressure in comparison with WT mice. (**D**) The upper airway was divided into 3 segments based on its surroundings and multilevel pressure drops (P_nasal_, P_hard_, and P_soft_) were assessed. (**E**) The rostral, caudal, and dorsal views of the upper airway are shown. The left-sided upper airway shown in blue is from a Dp16 mouse (female; 69-day-old; body weight is 18.6 g) and the right-sided one shown in red is from a WT mouse (female; 60-day-old; body weight is 19.1 g). The dotted line is set at the midline of the two and the size of the boxes surrounding the airway matches the upper airway of the WT mouse for ease of visual comparison. The Dp16 mouse has a dorsoventrally smaller and horizontally thinner nasal cavity (black arrows) and a shorter pharyngeal airway (double arrows). (**F**) The volume of the nasal cavity and pharyngeal airway is compared between genotypes. (**G**) Dp16 mice have a significantly shorter pharyngeal airway than WT mice. Values are presented as means ± SD. *P < 0.05; ^†^*P* < 0.001.

The maximum velocity (Vmax; m/s) and maximum negative pressure (Pmax; Pa) of the upper airway were estimated using 5.28 ml/s as a constant flow rate, which was the mean of peak inspiratory flow obtained with plethysmography. The Vmax in the nasal cavity did not differ between genotypes (WT = 12.26 ± 4.28 m/s, Dp16 = 14.76 ± 6.25 m/s, *P* = 0.369). On the other hand, the Vmax in the pharyngeal airway of Dp16 mice was significantly faster compared to that of WT mice (WT = 12.29 ± 1.16 m/s, Dp16 = 17.63 ± 2.63 m/s, *P* < 0.001) (Fig. 5A). Moreover, the velocity of air peaked at the narrowed area of the pharyngeal airway in Dp16 mice, while it was even throughout the upper airway in WT mice (Fig. 5B, arrow). Reflecting this faster velocity in the upper airway, Dp16 mice showed a significantly higher Pmax in comparison to WT mice (Fig. 5C; WT = − 689 ± 134 Pa, Dp16 = − 1085 ± 346 Pa, *P* = 0.015). In order to examine the contribution of upper airway segments, the inner pressures were reanalyzed in each segment (P_nasal_, P_hard_, and P_soft_). Although P_nasal_ accounted for the majority of the Pmax, a significantly higher negative pressure was observed in the hard palate and soft palate segments of Dp16 mice (Fig. 5D).

A 3D modeling representing each genotype was shown in Fig. 5E. The volume analysis demonstrated that the nasal cavity volume in Dp16 mice was significantly lower than in WT mice (WT = 18.56 ± 3.22 mm^3^, Dp16 = 14.49 ± 2.5 mm^3^, *P* = 0.020) (Fig. 5F). As represented in the 3D model, the volume reduction in the nasal cavity may be attributable to the dorsoventrally smaller and horizontally thinner nasal cavity rather than the longitudinal shortening (Fig. 5E, and Video S1 and S2). The comparison of the pharyngeal airway volume also showed significantly lower volume in Dp16 mice than in WT mice (WT = 5.13 ± 0.78 mm^3^, Dp16 = 3.39 ± 0.80 mm^3^, *P* < 0.001). This difference resulted from both the narrowing and shortening of the pharyngeal airway in Dp16 mice (WT = 5.80 ± 0.23 mm, Dp16 = 5.53 ± 0.15 mm, *P* = 0.014) (Figs. 3, 5E,G).

## Discussion

The high prevalence of OSA and difficulties with its management in children with DS has long been recognized. Although many mouse models replicating trisomy were developed and dozens of studies highlight molecular mechanisms and candidate genes for DS cognitive features, information regarding respiratory function and upper airway is limited. Here, we characterized the upper airway as well as craniofacial abnormalities in Dp16 mice.

A few morphological studies have reported using postmortem morphometric or micro-CT analysis of the skull of Ts65Dn and Dp16 mice^20–22^. However, unlike the nasal cavity or craniofacial bone structures, the upper airway would be well delineated in live mice because its structure and function are affected by serotonergic modulation and inner pressure during the respiratory cycle^19,23^. Thus, we optimized the imaging protocol and evaluated the craniofacial morphology of live Dp16 mice using a high-resolution micro-CT to further visualize upper airway structure and airflow dynamics. In general, the craniofacial anatomy of mice is characterized by a caudally elongated upper airway and its surrounding structures anatomically differ from those seen in humans. For example, the rostral part of the nasopharyngeal airway in mice is closely surrounded by bones^24,25^. Therefore, this rostral region can be directly affected by craniofacial abnormalities^25^. Our craniofacial measurements revealed characteristic craniofacial features of Dp16 mice; the mandible and the cranial bones surrounding the upper airway such as the maxilla, palatine, and presphenoid bones were significantly smaller compared to those of euploid mice. In accordance with the craniofacial manifestations, the upper airway of Dp16 mice was miniaturized in terms of size and length to fit the reduced space due to cranial base hypoplasia and fixed bony structure. Interestingly, micro-CT imaging revealed that the upper airway became narrowed and fluctuated in size more widely in the soft palate compartment than the hard palate compartment, suggesting that the upper airway surrounded by soft tissues would be more vulnerable to upper airway obstruction. Although one should be cautious in species differences in anatomy that could influence the nature of airflow, Dp16 mice would be potentially useful for subsequent airflow analysis.

CFD simulation has been used experimentally to describe pulmonary airflow dynamics and cardiovascular hemodynamics^26^. Some groups endeavor to apply CFD analysis to virtually predict and assess surgical treatment outcomes in OSA patients or DS patients with OSA^14,27–29^. Although this technique is still far from clinical practice due to large individual variability, the use of CFD analysis in well-validated Dp16 mice provides valuable insights in the airway structure and the site of obstruction by computing 3D modeling and flow parameters such as velocity and airflow pressure. With CFD analysis, we revealed that speed and pressure of air are strongly influenced by the narrowing of the pharynx in Dp16 mice. In addition, in combination with the morphological observation, 3D modeling thoroughly delineated the characteristics of the upper airway that Dp16 mice have a significantly smaller pharyngeal airway volume due to short length and small cross-sectional areas. On the contrary, the small nasal cavity would be less involved in the upper airway resistance in Dp16 mice. Despite the significant increase in the upper airway resistance, plethysmography showed little or no apparent differences in respiration during wakefulness between genotypes. This is most likely achieved by compensatory mechanisms as evidenced by increased respiratory efforts and relative prolongation of the inspiratory time to the expiratory time in Dp16 mice.

The Pmax value simulated at the epiglottis in this study is not equal to the critical closing pressure P_CRIT_, defined as the maximal nasal pressure at which the upper airway collapses. Since upper airway collapsibility is mainly determined by the passive structural factors (e.g., soft tissues and bony structures) and active neuromuscular control^30^, P_CRIT_ is categorized into three; passive P_CRIT_ and P_CRIT_ in the active condition during inspiration and expiration. We used the mean of peak inspiratory flow derived from plethysmography to estimate the maximal negative pressure, meaning that the estimated pressure could represent a pressure in the active condition during inspiration. According to a previous study, the mean of P_CRIT_ of the active condition during inspiration was reported as approximately -1400 Pa in C57BL/6J mice^30^. In the present study, the mean of Pmax in Dp16 mice was − 1085 Pa, while − 689 Pa in WT mice, implying that the margin to P_CRIT_ was smaller in Dp16 mice. Thus, the upper airway of Dp16 mice may be more susceptible to collapse especially in the presence of additional predisposing factors for OSA such as anesthesia, obesity, and abnormal muscular activity^19,31^. In fact, one of the Dp16 mice examined exhibited a upper airway collapse during micro-CT scanning, although this observation should be interpreted with caution because rescanning on the following day did not show a similar collapse.

A small upper airway does not always mean that it is more collapsible^32^. Nonetheless, upper airway narrowing is an important morphological characteristic seen in New Zealand obese mice^33^ and Zucker rats^34^, which are well-established rodents models demonstrating increased susceptibility to upper airway collapse. As seen in obese OSA patients, these models also have increased adipose tissue volume in pharyngeal structures including the tongue^33,35^. In contrast, Dp16 mice do not show spontaneous obesity but exhibit upper airway narrowing with severe craniofacial hypoplasia. Unfortunately, we could not measure the soft tissue volume in the craniofacial area due to technical limitations. It is worth considering the impact of macroglossia and soft tissue crowding, which are frequently seen in DS patients with persistent OSA^5,36^, because the narrowing is more prominent in the caudal part of the pharyngeal airway in Dp16 mice. In OSA patients, a caudally displaced hyoid bone is repeatedly reported as a marker of OSA severity and relative excessiveness of upper airway soft tissue for the craniofacial size^37–39^. On the contrary, the hyoid bone was positioned rostrally in Dp16 mice, presumably because of craniofacial hypoplasia. Interestingly, our preliminary data showed that there was no difference in the position of the hyoid bone between C57BL/6 mice on a regular diet and high-fat diet, suggesting that hyoid bone displacement is not likely a good marker of obesity in mice.

The sleep phenotype of Ts65Dn and Ts1Cje mice carrying one extra copy of partially overlapping segments of mouse chromosome 16, was reported in our previous study in which Ts65Dn mice showed increased waking amounts at the expense of non-REM sleep though Ts1Cje mice had no sleep or EEG abnormalities^40^. Recently, it was reported that aged Dp16 mice spend more time awake with the frequent transition from sleep to wakefulness and decreased delta power during non-REM sleep, similarly in sleep phenotype to Ts65Dn mice^41^. Although the occurrence of OSA has not been explored in these models so far, these findings are consistent with sleep disturbances found in individuals with DS. Sleep abnormalities in Dp16 mice may be, in part, explained as the manifestation of OSA in addition to altered neural activities.

Our study has several technical limitations as follows; (1) Micro-CT scanning was performed without synchronization to the respiratory cycle. It is known that both human subjects and obese mice show slight changes in dimensions of the upper airway throughout respiration^33,42,43^. However, in order to apply a respiratory-gated imaging protocol, we needed to sacrifice the resolution to shorten the exposure time per frame during the micro-CT scan, resulting in images at least 4 times coarser and a failure to generate a mouse-specific configuration of the upper airway, which is not optimal for CFD analysis. (2) Soft tissue volume was not measured due to the indistinct border between surrounding soft tissues. Therefore, we could not fully explore the possibility of macroglossia (3) The CFD results might be overestimated to some extent because the mean of peak inspiratory flow was obtained during wakefulness without movement while micro-CT images were acquired under anesthesia. Since this is the first report applying CFD analysis for the evaluation of upper airway in rodents, further studies are required to assess its feasibility and effectiveness in disease models. (4) Although the present study indicates an increased potential risk of OSA morbidity in Dp16 mice, respiratory problems in DS likely have multifactorial etiology such as obesity, ventilatory control stability, upper airway dilator muscle activity, and lung volume that were not characterized in this study. In addition, it is not clear how the findings are relevant to sleep abnormalities in Dp16 mice. Therefore, it is crucial to examine the occurrence of OSA-like events and respiratory function during sleep.

In conclusion, Dp16 mice demonstrated craniofacial hypoplasia, especially prominent in the ventral part of the skull and mandible, resulting in a significant multilevel narrowing of the upper airway. Furthermore, CFD revealed a high negative pressure inside the airway of Dp16 mice. These morphological and aerodynamic alterations can create an OSA-prone environment in Dp16 mice. Therefore, Dp16 mouse would be a potential model for studying sleep apnea in DS. Future studies such as on impact of obesity, soft-tissue volume, and OSA occurrence are required to assess the validity of this mouse model on DS sleep and breathing abnormalities.

## Methods

### Mouse model of DS

Dp16 and WT mice were obtained from The Jackson Laboratory (Bar Harbor, Maine, US). Each group was comprised of 8 mice (4 male and 4 female mice). The body weight was matched to minimize the sexual difference in growth rate, which potentially affects the size of structures. All procedures were approved by the Committee on the Ethics of Animal Experiments of the Stanford University Administrative Panel on Laboratory Animal Care, and complied with the USDA Animal Welfare Act.

### Whole-body plethysmography

To obtain baseline respiratory parameters during wakefulness, we used whole-body plethysmography. Unrestrained mice were placed inside a plethysmograph chamber (450 ml, Model PY4211; Buxco, DSI, Saint Paul, Minnesota, US) at room temperature in 21% O_2_ balanced with N_2_ and acclimated to the environment^44^. The values of the environmental condition such as room and chamber temperature and barometric pressure were manually inputted into IOX2 software (emka TECHNOLOGIES USA, Falls Church, Virginia, US). Spontaneous activity in the chamber was monitored by a video recorder. The average recording time was 26.8 ± 7.6 min, which was sufficient to obtain baseline respiratory parameters during wakefulness that was defined as no movement for at least 5 seconds based on the video. The following respiratory parameters were monitored and analyzed by IOX2: tidal volume, respiratory rate, minute volume, inspiratory time, expiratory time, peak inspiratory flow, and peak expiratory flow. To remove baseline and environmental noise from the data, the episodes were excluded if the calculated tidal volume was less than 0.05 ml or greater than 2.0 ml. Respiratory rate was calculated by dividing the number of breaths by the extracted time. Minute volume was calculated by multiplying tidal volume by respiratory rate.

### CT image acquisition

High-resolution micro-CT images were acquired using an in vivo micro-CT scanner SkyScan 1276 (Bruker, Billerica, Massachusetts, US) under isoflurane anesthesia. The scanning mode was set as 360°, step-and-shoot scanning without average framing. We applied voltage of 70 kV and a 0.5 mm aluminum filter, with the scan image voxel size of 20 µm and a binning of 2000. The exposure time per frame was 356 ms. This condition resulted in an average scan time of 19 min. The mice were free breathing and scanned prone on the animal bed with their heads gently fixed to the bed using an adhesive tape. In general, the decreased respiratory rate due to anesthesia causes the augmented respiratory motion, potentially resulting in large motion artifacts. Furthermore, given the depth of anesthesia can affect the collapsibility and neuromuscular activity of the upper airway^19^, a lighter depth of anesthesia is, theoretically, a better condition for upper airway imaging. Thus, we monitored their breathing (SkyScanVisual, Bruker) and maintained the respiratory rate at approximately 100 breathes per minute by adjusting the isoflurane dose. In addition, the original face mask on the animal bed required the upper pair of incisors to be hooked on an accessory, which opened the mouth and raised the neck. Therefore, we covered the face with a custom mask to maintain natural neck alignment. After each scan, the projection images were reconstructed using the software (NRecon with GPU acceleration, Bruker), followed by converting the set of reconstructed slices to DICOM files (DICOM converter, Bruker).

### Craniofacial and upper airway measurements

DICOM data were analyzed using Amira 6.7 Software (Thermo Fisher Scientific, Waltham, Massachusetts, US) for the craniofacial measurement and OsiriX 10.0 (Pixmeo, Geneva, Switzerland) for the upper airway measurements. Volume rendering was utilized for 3D visualization of each of the structures. Twenty-six landmarks were marked for craniofacial measurement and 2 additional landmarks were identified in the midline sagittal plane to evaluate the hyoid position (Figure S1)^21,22,45^. In total 26 linear distances were measured. One Dp16 mouse was excluded from the bone measurement due to hydrocephalus.

Multiplanar reconstruction (MPR) is a technique that generates sectional images in arbitrary planes, such as two-dimensional (2D) sagittal, coronal, and oblique views. Curved-MPR is a type of MPR available in OsiriX, which enables visualization of the whole length of a three-dimensionally tortuous structure within one single image and to reconstruct sectional images perpendicular to the structure. Thus, this method is especially useful in assessing the 2D profiles of a curved anatomic structure along its length. After selecting the “3D Curved-MPR” tool in Osirix, the central axis of the upper airway was manually defined, using the caudal edge of the hard palate as a landmark. Nine consecutive cross-sectional images perpendicular to the upper airway were obtained at 1 mm intervals, with the most rostral image being at 3 mm rostral to the landmark. Cross-sectional areas were measured manually by drawing the contour of the airway. Additionally, we evaluated the length of the pharyngeal airway from the caudal edge of the hard palate to the arytenoid cartilage in the 3D Curved-MPR plane.

### CFD modeling of the upper airway

The 3D volume images of the upper airway were generated using INTAGE Volume Editor (Cybernet, Tokyo, Japan)^46^. In order to embody and isolate the void airway space from surrounding tissues, conversion of a negative value to a positive value and vice versa was required, followed by adjusting an appropriate threshold to refine the airway region without losing a mouse-specific shape^46^. The data in stereolithographic format were exported to a CFD software, PHOENICS (CHAM-Japan, Tokyo, Japan), and fluid-mechanical simulation was performed. The flow was assumed to be steady, Newtonian, homogeneous, and incompressible^47^.

Airflow accelerates from the beginning of inspiration and reaches a peak flow rate around the middle of the inspiration cycle, generating the maximum negative pressure inside the airway. Thus, computational flow simulations using a peak inspiration flow allow us to estimate the highest negative pressure, which theoretically influences the airway collapse the most. The mean of peak inspiratory flows derived from plethysmography was used as a constant flow rate for all the mice so that the estimated pressure could reflect only the configuration of the upper airway. The upper airway from the nostrils to the epiglottis was divided into 3 segments based on its surroundings (the nasal cavity, hard palate, and soft palate). We used the following landmarks dividing the segments; (1) the merging of the right and left nasopharyngeal meatuses and (2) the caudal edge of the hard palate. Upper airway pressure was measured at the level of the epiglottis as well as the 2 landmarks with pressure at the nostrils set to 0 Pa. Pressure differences between 2 consecutive landmarks were represented for pressure drop at each segment (P_nasal_, P_hard_, and P_soft_). The airway volume was also measured from the nostrils to the point of 5 mm caudal to the distal edge of the hard palate.

### Statistical analysis

Statistical significance comparing the two groups was determined using the Welch’s *t* test. A *P* value less than 0.05 was considered statistically significant for the Welch’s *t* test. All statistical analyses were conducted using JMP 12.0 software (SAS Institute, Cary, North Carolina, US). Continuous variables are presented as mean and standard deviation (SD).

## Supplementary information

Supplementary Video Legends.

Video S1.

Video S2.

Supplementary Information.
