# Supplementary material for: Detailed evaluation of the upper airway in the Dp(16)1Yey mouse model of Down syndrome
Source: Sci Rep. 2020 Dec 7;10:21323. doi: 10.1038/s41598-020-78278-2 (PMC7721723; doi:10.1038/s41598-020-78278-2)
Supplement: Supplementary file 1 — Supplementary Video Legends. [file 41598_2020_78278_MOESM1_ESM.docx]

**Supplementary Materials for**

**Detailed evaluation of the upper airway in the Dp(16)1Yey mouse model of Down syndrome**

Tatsunori Takahashi ^a^, Noriaki Sakai ^a^*, Tomonori Iwasaki ^b^, Timothy C. Doyle ^c^, William C. Mobley ^d^, Seiji Nishino ^a^

^a^ Sleep and Circadian Neurobiology Laboratory, Department of Psychiatry and Behavioral Sciences, Stanford University School of Medicine

3155 Porter Drive, Room 2141, Palo Alto, CA 94304, USA

^b^ Department of Pediatric Dentistry, Kagoshima University Graduate School of Medical and Dental Sciences

8-35-1, Sakuragaoka, Kagoshima, Kagoshima 8908544, Japan

^c^ The Neuroscience Community Labs, Wu Tsai Neurosciences Institute, Stanford University

318 Campus Drive, Suite S170, Stanford, CA 94305, USA

^d^ Department of Neurosciences, University of California San Diego School of Medicine

9500 Gilman Drive, La Jolla, CA 92093, USA

**Online supplementary Video S1 and S2.**

Three-dimensional reconstruction of the upper airway. The left-sided upper airway shown in blue is from a Dp16 mouse and the right-sided one shown in red is from a WT mouse.
