# Supplementary material for: Detailed evaluation of the upper airway in the Dp(16)1Yey mouse model of Down syndrome
Source: Sci Rep. 2020 Dec 7;10:21323. doi: 10.1038/s41598-020-78278-2 (PMC7721723; doi:10.1038/s41598-020-78278-2)
Supplement: Supplementary file 4 — Supplementary Information. [file 41598_2020_78278_MOESM4_ESM.docx]

**Figure S1. Three-dimensional reconstruction of micro-CT images of a mouse skull and landmarks**

The dorsal (A), ventral (B), and rostral (C) views of the cranium and the lateral (D) view of the mandible are shown. For analysis of the hyoid position, the midline sagittal plane was used (E). Craniofacial landmarks are as follows (number, definition) and right-sided landmarks are shown in parentheses: 1, nasale; 2 and (4), intersection of frontal, premaxilla and maxilla bones; 3, nasion; 5 and (7), most prominent lateral point on frontal-parietal suture; 6, bregma; 8 and (10), intersection of parietal, interparietal and occipital bones; 9, lambda; 12 and (11), anterior-most point of anterior palatine foramen; 13, medial premaxillary-maxillary junction; 14, midline point on maxillary-palatal suture; 16 and (15), posterior-most point on the central anteroposterior axis of molar alveolus; 17, posterior point on palate; 18, anterior point at the base of nasal aperture; 19 and (20), superior-most point on incisor alveolar rim (at bone-tooth junction); 21 and (22), inferior-most point on border of ramus inferior to incisor alveolar; 23 and (24), apex of coronoid process; 25 and (26), posterior-most point on mandibular condyle; 27, ventral point on basisphenoid-occipital suture at midline; 28, hyoid bone at midline.

**Supplemental Table. Comparisons of craniofacial measurements between WT and Dp16 mice according to sex**

|  | Landmarks (Suppl. Figure 1) |  | Male | | | | | |  | Female | | | | | |
| --- | --- | --- | --- | --- | --- | --- | --- | --- | --- | --- | --- | --- | --- | --- | --- |
|  |  |  | WT (N=4) | |  | Dp16 (N=4) | |  |  | WT (N=4) | |  | Dp16 (N=3) | |  |
|  |  |  | Mean | SD |  | Mean | SD | P |  | Mean | SD |  | Mean | SD | P |
| Skull (Figure 2A-C) | |  |  |  |  |  |  |  |  |  |  |  |  |  |  |
| Dorsal view |  |  |  |  |  |  |  |  |  |  |  |  |  |  |  |
| a | 1-3 |  | 7.14 | 0.16 |  | 7.05 | 0.24 | 0.593 |  | 7.04 | 0.25 |  | 7.00 | 0.15 | 0.801 |
| b | 3-6 |  | 7.89 | 0.17 |  | 7.40 | 0.11 | < 0.001 |  | 7.98 | 0.19 |  | 7.23 | 0.27 | 0.021 |
| c | 6-9 |  | 3.80 | 0.21 |  | 3.79 | 0.29 | 0.989 |  | 3.84 | 0.09 |  | 3.48 | 0.01 | 0.004 |
| d | 2-4 |  | 4.20 | 0.02 |  | 3.78 | 0.02 | < 0.001 |  | 4.19 | 0.04 |  | 3.74 | 0.03 | < 0.001 |
| e | 5-7 |  | 5.95 | 0.09 |  | 6.06 | 0.10 | 0.143 |  | 6.01 | 0.27 |  | 5.91 | 0.21 | 0.619 |
| f | 8-10 |  | 7.92 | 0.16 |  | 7.64 | 0.30 | 0.163 |  | 7.84 | 0.24 |  | 7.39 | 0.20 | 0.046 |
|  |  |  |  |  |  |  |  |  |  |  |  |  |  |  |  |
| Ventral view | |  |  |  |  |  |  |  |  |  |  |  |  |  |  |
| g | 11-15 |  | 7.43 | 0.17 |  | 6.85 | 0.07 | 0.003 |  | 7.38 | 0.20 |  | 6.82 | 0.08 | 0.006 |
| h | 12-16 |  | 7.45 | 0.16 |  | 6.78 | 0.11 | < 0.001 |  | 7.39 | 0.23 |  | 6.81 | 0.07 | 0.011 |
| i | 15-16 |  | 3.39 | 0.03 |  | 3.17 | 0.01 | < 0.001 |  | 3.41 | 0.06 |  | 3.21 | 0.13 | 0.040 |
| j | 13-18 |  | 5.27 | 0.15 |  | 4.73 | 0.10 | 0.001 |  | 5.27 | 0.14 |  | 4.76 | 0.39 | 0.072 |
| k | 13-14 |  | 3.81 | 0.11 |  | 3.42 | 0.09 | 0.002 |  | 3.69 | 0.10 |  | 3.32 | 0.30 | 0.089 |
| l | 14-17 |  | 2.13 | 0.05 |  | 1.95 | 0.05 | 0.002 |  | 2.09 | 0.04 |  | 1.89 | 0.11 | 0.025 |
|  |  |  |  |  |  |  |  |  |  |  |  |  |  |  |  |
| Lateral view | |  |  |  |  |  |  |  |  |  |  |  |  |  |  |
| m | 1-18 |  | 2.70 | 0.10 |  | 2.55 | 0.05 | 0.046 |  | 2.62 | 0.07 |  | 2.48 | 0.03 | 0.025 |
| n | 3-13 |  | 4.78 | 0.13 |  | 4.59 | 0.14 | 0.091 |  | 4.78 | 0.11 |  | 4.75 | 0.17 | 0.814 |
|  |  |  |  |  |  |  |  |  |  |  |  |  |  |  |  |
| Mandible (Figure 2D-E) | |  |  |  |  |  |  |  |  |  |  |  |  |  |  |
| o | 19-21 |  | 5.17 | 0.10 |  | 4.81 | 0.05 | 0.002 |  | 5.04 | 0.07 |  | 4.69 | 0.20 | 0.033 |
| p | 20-22 |  | 5.11 | 0.15 |  | 4.65 | 0.14 | 0.004 |  | 5.09 | 0.15 |  | 4.66 | 0.18 | 0.010 |
| q | 21-23 |  | 6.87 | 0.11 |  | 6.37 | 0.07 | < 0.001 |  | 6.84 | 0.16 |  | 6.56 | 0.21 | 0.084 |
| r | 22-24 |  | 6.92 | 0.10 |  | 6.49 | 0.18 | 0.012 |  | 6.92 | 0.15 |  | 6.57 | 0.19 | 0.027 |
| s | 19-23 |  | 8.61 | 0.11 |  | 7.89 | 0.12 | < 0.001 |  | 8.53 | 0.11 |  | 7.96 | 0.26 | 0.015 |
| t | 20-24 |  | 8.56 | 0.12 |  | 7.90 | 0.17 | 0.001 |  | 8.58 | 0.26 |  | 8.02 | 0.25 | 0.023 |
| u | 23-25 |  | 3.87 | 0.17 |  | 3.85 | 0.19 | 0.895 |  | 3.99 | 0.10 |  | 3.86 | 0.17 | 0.247 |
| v | 24-26 |  | 3.86 | 0.18 |  | 3.83 | 0.18 | 0.805 |  | 3.88 | 0.05 |  | 3.87 | 0.22 | 0.900 |
| w | 23-24 |  | 9.51 | 0.06 |  | 9.08 | 0.35 | 0.092 |  | 9.41 | 0.18 |  | 9.03 | 0.08 | 0.017 |
|  |  |  |  |  |  |  |  |  |  |  |  |  |  |  |  |
| Hyoid position (Figure 2C) | |  |  |  |  |  |  |  |  |  |  |  |  |  |  |
| x | 17-28 |  | 5.08 | 0.12 |  | 4.71 | 0.09 | 0.004 |  | 4.91 | 0.05 |  | 4.69 | 0.05 | 0.001 |
| y | 17-27 |  | 4.25 | 0.16 |  | 3.36 | 0.17 | < 0.001 |  | 4.12 | 0.11 |  | 3.31 | 0.23 | 0.002 |
| z | 27-28 |  | 2.78 | 0.13 |  | 2.83 | 0.03 | 0.450 |  | 2.80 | 0.13 |  | 2.74 | 0.19 | 0.635 |

The unit is millimeter.
